# Buffalo Colleges

**Published:** 1887-12

**Authors:** 


### Editorial.

### BUFFALO COLLEGES.

The regular announcements of the opening of our Colleges has been crowded out of former numbers. The Buffalo University opened September 20th with appropriate exercises. The address of the evening was made by Dr. Henry R. Hopkins, which was well received by the large class and their friends who were present. The College has a fine class in attendance, numbering about 120 in the Medical Department, and thirty-five in the

School of Pharmacy. The new Professor of Theory and Practice, Prof. Chas. G. Stockton, fully meets, it is said, the most sanguine expectations of his many friends, who congratulated the College on this commendable appointment. The recovery of Prof. Mann is a source of congratulation to the College as well as the general profession. Dr. John H. Pryor, of this city, has been appointed Adjunct-Professor of Materia Medica, Therapeutics and Hygiene, and, it is generally understood, will give most of the lectures on this branch. Dr. Van Den Bergh, who has been an associate of Prof. Withaus for the past year, will give most of the lectures and instruction in Chemistry, Dr. Withaus now being in New York permanently. The College of Pharmacy faculty remains the same, and the course is under the direction of Dr. Van Den Bergh, Dr. Gregory, and Prof. Kellicott, of the Normal School. Prof. Oldberg, of Chicago, will be in attendance two weeks of the course, and give the usual number of lectures during that time.

Niagara University was opened one day later, and has a large class of well-educated young men in attendance, numbering, in all, about fifty in the Medical Department, and about twenty in the Law School. The opening address of the Medical School was given by Prof. A. A. Hubbell, of the faculty, who took for his subject the aims of the medical student. He commented at length on the following:

1st.—Motives which actuated them in entering upon the study of medicine, and those which are to be commended.

2d.—The duties and objects of the physician's vocation, and the factors that will contribute to his success; primarily, a thorough medical training, involving faithful and prolonged study, as well as a careful preliminary preparation for the study.

3d.—Some of the achievements of American medicine in the past, and the outlook and prospects for its future, and the encouragement which should stimulate the student of to-day to study and work, and make a history and name for himself as worthy and renowned as any in the past.

The address was instructive, and listened to with marked interest throughout.

The course is progressing satisfactorily. The Maternity Hospital, which is conducted by Dr. Lothrop, affords each member of the graduating class an opportunity to attend two or three accouchements, under the direction of Dr. Frederick, the assistant in Obstetrics, thereby affording facilities in this branch enjoyed by few colleges in this country. The Dispensary, the Emergency Hospital, and the Hospital of the Sisters of Charity afford ample clinical material. The clinical lectures of Prof. Cronyn, in the wards of the hospital, receive many expressions of praise from the students.

By the promotion of Dr. Campbell to the chair of Materia Medica and Therapeutics, it has already been demonstrated, the faculty and trustees made a wise choice. The Doctor delights, as well as instructs, his students, and, without a dissenting voice, he is accorded the highest rank as a lecturer and teacher. The chair of Hygiene has been filled by a most estimable man, and this branch, which is assuming such great proportions in the medical science, will, doubtless, be well taught by Dr. A. E. Persons, who has been appointed lecturer on this subject. The Doctor has had a classical education and a thorough medical training; was graduated from the New York College of Physicians and Surgeons. He has practiced about six years, four of them being in this city.

The Law School is well attended, there being, as stated, twenty students. This far excels the expectations of its founders, and augurs well for the future of the College. The opening exercises were held October 3d, the address being given by Hon. Chas. Daniels, LL.D., Dean of the faculty. The lecturers in all departments are well chosen, as the experience of the past two months demonstrates.

